# Food security reduces multiple HIV infection risks for high‐vulnerability adolescent mothers and non‐mothers in South Africa: a cross‐sectional study

**DOI:** 10.1002/jia2.25928

**Published:** 2022-08-25

**Authors:** Lucie Cluver, William E. Rudgard, Elona Toska, Mark Orkin, Mona Ibrahim, Nontokozo Langwenya, Caroline Kuo, Nonhlanhla Xaba, Kai Roehm, Michael Smith, Sara Bernardini, Giovanni Giordana, Manaan Mumma, James Kingori, Rachel Yates, Lorraine Sherr

**Affiliations:** ^1^ Department of Social Policy and Intervention University of Oxford Oxford UK; ^2^ Department of Psychiatry and Mental Health University of Cape Town Cape Town South Africa; ^3^ Centre for Social Science Research University of Cape Town Cape Town South Africa; ^4^ Medical Research Council Development Pathways to Health Research Unit, School of Clinical Medicine University of the Witwatersrand Johannesburg South Africa; ^5^ Department of Behavioral and Social Sciences, School of Public Health Brown University Providence Rhode Island USA; ^6^ UN World Food Programme, Regional Bureau for Southern Africa Johannesburg South Africa; ^7^ UN World Food Programme Rome Italy; ^8^ UN World Food Programme Regional Bureau for Eastern Africa Nairobi Kenya; ^9^ Health Psychology Unit, Institute of Global Health University College London London UK

**Keywords:** adolescent girls and young women, motherhood, risky behaviour, food security, HIV, South Africa

## Abstract

**Introduction:**

Adolescent girls and young women, including adolescent mothers, in Southern Africa have high HIV seroconversion and transmission. We need to know which risks drive HIV infections, and what can reduce these risks.

**Methods:**

We interviewed 1712 adolescent girls and young women (11–23 years), including 1024 adolescent mothers who had conceived before age 20 and had a living child, from two health municipalities of South Africa's Eastern Cape Province between March 2018 and July 2019. Recruitment was through multiple community, school and health facility channels. Associations between adolescent motherhood and seven HIV risk behaviours (multiple sexual partners, transactional sex, age‐disparate sex, condomless sex, sex on substances, alcohol use and not in education or employment) were investigated using the generalized estimating equations method for multiple outcomes specified with a logit link and adjusting for nine covariates. Using the same model, we investigated associations between having enough food at home every day in the past week (food security) and the same seven HIV risk behaviours. When we found evidence of moderation by HIV status, we report stratum‐specific odds ratios.

**Results:**

Mean age was 17.51 years (SD: 2.54), 46% participants were living with HIV. Compared to non‐mothers, adolescent mothers had lower odds of alcohol use (AOR = 0.47, 95% CI = 0.29–0.75), but higher odds of multiple sexual partners (AOR = 1.93, 95% CI = 1.35–2.74), age‐disparate sex (HIV‐uninfected AOR = 1.73, 95% CI = 1.03–2.91; living with HIV AOR = 5.10, 95% CI = 2.98–8.73), condomless sex (AOR = 8.20, 95% CI = 6.03–11.13), sex on substances (AOR = 1.88, 95% CI = 1.10–3.21) and not in education/employment (HIV‐uninfected AOR = 1.83, 95% CI = 1.19–2.83; living with HIV AOR = 6.30, 95% CI = 4.09–9.69). Among non‐mothers, food security was associated with lower odds of multiple sexual partners (AOR = 0.45, 95% CI = 0.26–0.78), transactional sex (AOR = 0.32, 95% CI = 0.13–0.82) and not in education/employment (AOR = 0.48, 95% CI = 0.29–0.77). Among adolescent mothers, food security was associated with lower odds of transactional sex (AOR = 0.17, 95% CI = 0.10–0.28), age‐disparate sex (AOR = 0.66, 95% CI = 0.47–0.92), sex on substances (AOR = 0.51, 95% CI = 0.32–0.82), alcohol use (AOR = 0.45, 95% CI = 0.25–0.79) and not in education/employment (AOR = 0.56, 95% CI = 0.40–0.78).

**Conclusions:**

Adolescent motherhood is associated with multiple vulnerabilities to HIV infection and transmission. Social protection measures that increase food security are likely to reduce HIV risk pathways for adolescent girls and young women, especially adolescent mothers.

## 1 INTRODUCTION

In Southern and Eastern Africa, adolescent girls and young women (AGYW) are more than twice as likely to acquire HIV than their male peers [1], and around 20% become pregnant before turning 20 [2, 3, 4]. AGYW who are pregnant or mothers experience significantly higher risk of HIV infection than their peers [5, 6, 7, 8]. Additionally, although antenatal care commonly serves as a critical entry point for HIV testing, low rates of disclosure to partners and use of maternal anti‐retroviral therapy (ART) is a risk for onwards HIV transmission to children and future sexual partners [9, 10, 11, 12, 13].

There is still limited evidence of the risk factors for HIV infection during adolescent pregnancy and motherhood. Like for horizontally acquired HIV, adolescent motherhood is associated with higher rates of age‐disparate sex and multiple sexual partners [6, 7]. Furthermore, many of the structural drivers of HIV infection, including exposure to poverty, gender inequality and violence, are also associated with adolescent pregnancy and motherhood [14, 15]. It is also possible that hormonal changes contribute to higher rates of HIV infection during pregnancy [16, 17]. Presently, with so few studies in this area, further research is needed, particularly focusing on the relationship between adolescent motherhood and other known HIV risk behaviours, such transactional sexual relationships [18], substance use‐related risks [19], reduced capacity to negotiate protected sex [20] and school dropout [21].

In addition to understanding the drivers of heightened risk of HIV infection, we also need evidence on effective interventions for HIV prevention among adolescent mothers. This has become even more critical since Covid‐19‐related disruptions to family planning services are expected to have led to a significant rise in unplanned pregnancies [22, 23]. Social protection, using the UN interagency definition of “policies and programmes aimed at preventing, and protecting people against, poverty, vulnerability and social exclusion throughout their life, with a particular emphasis on vulnerable groups,” is endorsed as a critical enabler of HIV prevention among AGYW [24, 25, 26, 27, 28]. Substantial evidence shows that in contexts of high poverty, cash and in‐kind transfers are effective for enhancing food security, which is a key determinant of AGYW’s economic dependency on men and engagement in HIV risk behaviours, like transactional sex [29, 30].

In this study, we aimed to assess if adolescent mothers in South Africa are at heightened risk of seven HIV risk behaviours and investigate the potential of food security to simultaneously reduce multiple risk pathways to HIV infection in both adolescent mothers and non‐mothers.

## 2 METHODS

### 2.1 Study design

The study used a cross‐sectional design and was reported according to the Strengthening the Reporting of Observational Studies in Epidemiology checklist for cross‐sectional studies (Table S1) [31].

### 2.2 Study setting

The study site was 180 communities spread across Amathole district and Buffalo City metropolitan municipalities of South Africa's Eastern Cape Province. Interviews took place between March 2018 and July 2019. The Eastern Cape is one of the poorest provinces in South Africa with a Human Development Index around 0.67 [32]. The 2017 South African National HIV Prevalence, Incidence, Behaviour, and Communication Survey estimated that 10% of AGYWs in the province were living with HIV [33].

### 2.3 Study sample

The study population was AGYW (11–25 years) without a live‐born child (non‐mothers), and AGYW who had conceived before age 20 and had a living child from that pregnancy (adolescent mothers). The study sample included AGYW participating in the Mzantsi Wakho (MW) cohort studying the lived experience of adolescents living with HIV [34], and the HEY BABY cohort studying resilience among adolescent parent families [35]. Recruitment for MW occured between March 2014 and September 2015. All adolescents (10–19 years) who had ever initiated HIV care in one of the 73 health facilities providing ART across the study site were contacted and invited to participate in the study. To prevent stigmatization of adolescents living with HIV, cohabiting or neighbouring adolescents were also invited to participate [34]. The study had 90% uptake at baseline and 94% retention at follow‐up interviews in 2018/2019. Recruitment for HEY BABY occured between March 2018 and July 2019 and used multiple channels that were developed with an advisory group of adolescent mothers [35]. First, all adolescent mothers in the MW cohort were invited to participate. Then, we contacted and invited participants using patient files at ART clinics and maternity obstetric units across the study site and teacher referrals at 43/149 randomly selected secondary schools. There was also community recruitment via door‐to‐door visits and referrals from community guides. Finally, to ensure recruitment of the most vulnerable adolescent mothers, we used referrals from local social workers, NGOs, and adolescent mothers themselves.

### 2.4 Ethics

Ethical approvals were obtained from the University of Oxford (R48876/RE001,SSD/CUREC2/12‐21), University of Cape Town (HREC 226/2017,CSSR 2013/4), Provincial Departments of Health and Basic Education, health facilities and schools. All adolescents and all primary caregivers (where adolescents were under 18 years old) gave voluntary informed consent, read aloud in cases of low literacy. There were no financial incentives for participation, but adolescents received a certificate and small gift pack, including toiletries for girls and babies. Interviews were conducted in a location of the adolescent's choice and took 45–70 minutes. They used audio mobile‐assisted self‐interviewing on electronic tablets, assisted by local interviewers trained to adjust level of assistance by age, literacy and confidence of participants. Interviews took place in Xhosa or English, according to participant choice. Confidentiality was maintained except when participants disclosed serious risk of harm to themselves or others. In these cases, safeguarding processes were followed. For reports of current abuse, recent rape or suicidality, participants were immediately supported to access post‐exposure prophylaxis, pregnancy prevention and child protection measures with government or NGO services. Findings of the study are shared with communities, health facilities and government in research areas as part of embedded local knowledge sharing.

#### 2.4.1 Measures

Measures and scales were pre‐piloted with 34 local adolescents, including adolescent mothers. Input to questionnaire design was given by the South African National Departments of Health, Basic Education, and Social Development, the South African National AIDS Council, UNICEF, PEPFAR, USAID and local NGOs. All questionnaires are available at www.youngcarers.org.za.

#### 2.4.2 HIV infection risks

We assessed seven high‐risk behaviours for HIV infection and transmission, using questions adapted from the National Survey of HIV and Risk Behaviour among young South Africans, the PREPARE trial and the Child Behaviour Checklist Youth Self‐Report [36] (all self‐report): (1) *Multiple sexual partners*, measured as 2+ sexual partners in the past year [37], (2) *Transactional sex*, measured as past‐year receipt of money, drinks, clothes, airtime, a place to stay, lifts in a car/taxi, better marks at school, school fees, food or other material exchange for having sex; (3) *Age disparate sex*, measured as a sexual partner more than 5 years older in the past year; (4) *Condomless sex*, measured as ever not using a condom for the duration of sex in the past year; (5) *Sex on substances*, measured as having sex when drunk or using drugs in the past year; (6) *Alcohol use*, measured as responding “somewhat true” or “definitely yes” to the question “I drink alcohol to have a good time, without my caregivers knowing or approving in the past six months” [38], (7) *Not in education or employment*, measured as non‐enrolment in primary school, secondary school, university, college, further education and training, and not currently being paid a salary/wage full‐time or part‐time at the time of interview.

#### 2.4.3 Adolescent motherhood

Measured as conception of first child before age 20 according to the World Health Organization's definition [39]. Mothers’ age at conception was calculated by subtracting a conservative estimate of 294 days from first child's date of birth, and comparing this to mother's date of birth.

#### 2.4.4 Food security

Measured as having enough food at home every day in the previous seven days, using the South African National Food Consumption Survey food frequency questionnaire, adapted in pre‐piloting with local adolescents [40].

#### 2.4.5 Covariates

We considered nine covariates: participant HIV status, age, relationship status, parental monitoring, rural/urban household location, number of household residents, informal/shack housing, maternal orphanhood and paternal orphanhood. HIV status was assessed using clinical files for all participants recruited from health facilities. For girls and women not recruited via a health facility, HIV status was measured by participant self‐report during a series of semi‐structured questions by trained research assistants at the beginning of each interview, and confirmed in medical records where possible. For adolescent mothers, we cross‐checked self‐reported HIV statuses with data extracted from participants’ Road To Health card (a routine patient‐held medical record summarizing a child's health in the first 5 years of life). Parental monitoring was assessed using relevant items from the youth self‐report form of the Alabama Parenting Questionnaire [41]. Being in a relationship was measured as reporting a current boyfriend/girlfriend or being married. Number of household residents considered individuals living in a home for four or more nights per week.

#### 2.4.6 Data analysis

The analysis was carried out in seven stages [42]. We first investigated the relationship between HIV risk behaviours and adolescent motherhood. For this, we described the characteristics of participants and prevalence of seven HIV risk behaviours overall and by adolescent motherhood status. Second, we investigated the prevalence of HIV risk behaviours among non‐mothers and adolescent mothers by HIV status. Third, we used the generalized estimating equations (GEE) method for multiple outcomes specified with a logit link, to simultaneously model associations between adolescent motherhood and our seven HIV risk behaviours [43]. We used the GEE approach as (1) multiple outcomes were clustered within individuals, (2) interest lay in the fixed parameters of the model and (3) the GEE approach is more robust than a random intercept model to misspecification of the covariance structure between multiple outcomes [44]. The model controlled for food security and all nine additional covariates to reduce the risk of confounding bias and increase precision, and it was specified with an unstructured covariance structure as we had no theoretical justification that outcomes would be correlated equally [43]. Fourth, using the same model, we tested if relationships between motherhood and HIV risk behaviours were moderated by HIV status. Fifth, we investigated the relationship between food security and HIV risk behaviours, stratified by adolescent motherhood. Sixth, we tested if relationships between food security and HIV risk behaviours were moderated by HIV status. Seventh, still stratified by adolescent motherhood, we calculated adjusted probabilities and adjusted probability differences comparing the two scenarios: “not experiencing food security” and “experiencing food security.” All analyses were conducted in Stata 15 and missing values were handled by listwise deletion.

#### 2.4.7 Sensitivity analysis

We investigated the impact of controlling for sexual debut in our analyses. This served to identify which associations may be related to higher rates of first sexual experience in adolescent mothers as compared to non‐mothers.

## 3 RESULTS

We interviewed 1712 AGYW. Twenty‐two mothers were excluded due to conception after age 19 (Figure S1). Of 1690 participants included, 61% were adolescent mothers who had conceived their first child before age 20, with an average age of conception of 16.29 years (SD: 1.59). Missing values were <2% for all variables (Table S2).

### 3.1 Descriptive data

Characteristics of non‐mothers and adolescent mothers are summarized in Table 1. The average age of the sample was 17.51 years (SD: 2.54 years), 46% of participants were living with HIV, 29% were maternally orphaned and 31% were paternally orphaned. Mean household size was 6.15 (SD: 2.82). Fifty‐four percent of girls were in a relationship, but <1% reported being married. Twenty‐seven percent lived in rural areas and 19% in informal (shack) housing. Compared to non‐mothers, adolescent mothers were less likely to be living with HIV (*p* < 0.001), maternally orphaned (*p* < 0.001), or paternally orphaned (*p* = 0.001). Adolescent mothers were also older (*p* < 0.001), lived in larger households (*p* = 0.005) and were more likely to be in a relationship (*p* < 0.001). Past‐week food security did not differ by adolescent motherhood status.

**Table 1:** Characteristics of study participants and study outcomes overall and by adolescent motherhood

	Overall *N* = 1690 *n* (%)	Non‐mothers *N* = 666 *n* (%)	Adolescent mothers *N* = 1024 *n* (%)	*p*‐Value
Characteristics				
Age	17.51 (2.54)	16.39 (3.05)	18.24 (1.79)	<0.001
HIV status				<0.001
Living with HIV	771 (46)	480 (72)	291 (28)	
Relationship status				<0.001
In a relationship^a^	907 (54)	221 (33)	686 (67)	
Parental monitoring^b^	6.88 (8.39)	5.39 (7.30)	7.84 (8.90)	<0.001
Rural				0.1
Yes	459 (27)	166 (25)	293 (29)	
Informal house				<0.001
Yes	312 (18)	88 (13)	224 (22)	
Household size	6.15 (2.82)	5.92 (3.04)	6.31 (2.66)	0.005
Maternal orphan				<0.001
Yes	493 (29)	294 (44)	199 (19)	
Paternal orphan				0.001
Yes	522 (31)	235 (35)	287 (28)	
Food security				0.12
Yes	1260 (75)	510 (77)	750 (73)	
HIV risk behaviours				
Multiple sexual partners				<0.001
Yes	350 (21)	80 (12)	270 (26)	
Transactional sex				<0.001
Yes	100 (6)	21 (3)	79 (8)	
Age‐disparate sex^c^				<0.001
Yes	283 (17)	47 (7)	236 (23)	
Condomless sex				<0.001
Yes	761 (45)	90 (14)	671 (66)	
Sex on substances				<0.001
Yes	127 (8)	26 (4)	101 (10)	
Alcohol use				0.081
Yes	117 (7)	55 (8)	62 (6)	
Not in education/employment				<0.001
Yes	572 (34)	110 (17)	462 (45)	

Note: Data are mean (SD) for continuous variables and *n* (%) for categorical variables.

Abbreviations: HIV, human immunodeficiency virus; SD, standard deviation.

^a^ Measured as reporting a boyfriend/girlfriend or being married. Among participants *in a relationship*, 14 were married.

^b^Higher scores correspond with worse parental monitoring.

^c^ 24 participants are missing information on age‐disparate sex, including five non‐mothers and 19 adolescent mothers.

**Figure 1:**
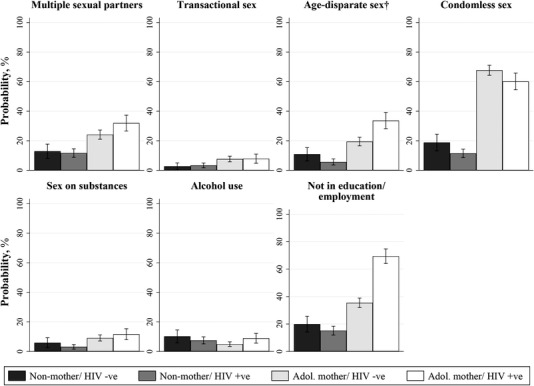
Risk of seven HIV risk behaviours by adolescent motherhood and HIV status. Note: *N* = 1690. †24 participants are missing information on age‐disparate sex, including five non‐mothers and 19 adolescent mothers. Abbreviation: HIV, human immunodeficiency virus.

Prevalence of studied HIV risk behaviours is summarized in Table 1. Among all AGYW, the most common risks were condomless sex (45%), not in education/employment (34%), multiple sexual partners (21%) and age‐disparate sex (17%); followed by sex on substances (8%), alcohol use (7%) and transactional sex (6%). Compared to non‐mothers, adolescent mothers reported significantly higher rates of all HIV risk behaviours except alcohol use (Table 1). Stratifying further by HIV status, rates of age‐disparate sex and not in education/employment were significantly higher among adolescent mothers living with HIV as compared to adolescent mothers not living with HIV (Figure 1). Correlations between study outcomes are reported separately for non‐mothers and adolescent mothers in Table S3, and univariable associations between adolescent motherhood and HIV risk behaviours are reported in Table S4.

### 3.2 Multivariate multivariable associations between adolescent motherhood and HIV risk behaviours

We found evidence that HIV status moderated the association between adolescent motherhood and age‐disparate sex (*p* = 0.003) and not in education/employment (*p* < 0.001) (Table 2). Adolescent motherhood was associated with lower odds of alcohol use (AOR = 0.47, 95% CI = 0.29–0.75) and higher odds of five out of the six other HIV risk behaviours: multiple sexual partners (AOR = 1.93, 95% CI = 1.36–2.74), age‐disparate sex (HIV‐uninfected AOR = 1.73, 95% CI = 1.03–2.91; living with HIV AOR = 5.10, 95% CI = 2.98–8.73), condomless sex (AOR = 8.20, 95% CI = 6.03–11.13), sex on substances (AOR = 1.88, 95% CI = 1.10–3.21) and not in education/employment (HIV‐uninfected AOR = 1.83, 95% CI = 1.19–2.83; living with HIV AOR = 6.30, 95% CI = 4.09–9.69). Although adolescent motherhood was also associated with higher odds of transactional sex, this was not statistically significant at the 5% level (*p* = 0.057).

**Table 2:** Multivariate multivariable associations between adolescent motherhood and HIV risk behaviours overall and stratified by HIV status

	Multiple sexual partners		Transactional sex		Age‐disparate sex		Condomless sex	
	AOR (95% CI)	*p*‐value	AOR (95% CI)	*p*‐value	AOR (95% CI)	*p*‐value	AOR (95% CI)	*p*‐value
Adolescent motherhood								
Overall	1.93 (1.36–2.74)	<0.001	1.82 (0.98–3.39)	0.057	3.26 (2.13–4.98)	<0.001	8.20 (6.03–11.13)	<0.001
Among HIV‐uninfected AGYW	2.07 (1.23–3.48)	0.706	2.34 (0.93–5.90)	0.484	1.73 (1.03–2.91)	0.003	9.11 (6.05–13.70)	0.434
Among AGYW living with HIV	1.81 (1.14–2.88)		1.53 (0.67–3.51)		5.10 (2.98–8.73)		7.24 (4.71–11.14)	

	Sex on substances		Alcohol use		Not in education/employment			
	AOR (95% CI)	*p*‐value	AOR (95% CI)	*p*‐value	AOR (95% CI)	*p*‐value		
Adolescent motherhood								
Overall	1.88 (1.10–3.21)	0.020	0.47 (0.29–0.75)	0.001	3.74 (2.67–5.23)	<0.001		
Among HIV‐uninfected AGYW	1.60 (0.78–3.29)	0.566	0.40 (0.21–0.77)	0.494	1.83 (1.19–2.83)	<0.001		
Among AGYW living with HIV	2.16 (1.03–4.51)		0.54 (0.30–0.99)		6.30 (4.09–9.69)			

Note: *n* = 1638. For sub‐group analyses, we report Wald test *p*‐values for the interaction term. Multivariable models adjust for participant characteristics: HIV status; age; relationship status; parental monitoring; rural/urban household location; informal housing type; number of people living in household; maternal orphanhood; paternal orphanhood; and food security. Clustering of multiple outcomes within individuals is accounted for using the GEE method.

Abbreviations: AGYW, adolescent girls and young women; AOR, adjusted odds ratio; CI, confidence interval; HIV, human immunodeficiency virus.

### 3.3 Multivariate multivariable associations between food security and HIV risk behaviours among non‐mothers and adolescent mothers

Across the seven HIV risk behaviours, we found evidence that the relationship between household food security and age‐disparate sex was moderated by adolescent motherhood (*p* = 0.037) (Table 3). We found no evidence that the association between food security and HIV risk behaviours was further moderated by HIV status (Table 3). Among non‐mothers, household food security was associated with lower odds of three out of the seven HIV risk behaviours: multiple sexual partners (AOR = 0.45, 95% CI = 0.26–0.78), transactional sex (AOR = 0.32, 95% CI = 0.13–0.82) and not in education/employment (AOR = 0.48, 95% CI = 0.29–0.77). Among adolescent mothers, household food security was associated with lower odds of five of the seven HIV risk behaviours: transactional sex (AOR = 0.17, 95% CI = 0.10–0.28), age‐disparate sex (AOR = 0.66, 95% CI = 0.47–0.92), sex on substances (AOR = 0.51, 95% CI = 0.32–0.82), alcohol use (AOR = 0.45, 95% CI = 0.25–0.79) and not in education/employment (AOR = 0.56, 95% CI = 0.40–0.78).

**Table 3:** Multivariate multivariable associations between food security and HIV risk behaviours overall and stratified by adolescent motherhood, and both adolescent motherhood and HIV status

	Multiple sexual partners		Transactional sex		Age‐disparate sex		Condomless sex	
	AOR (95% CI)	*p*‐value	AOR (95% CI)	*p*‐value	AOR (95% CI)	*p*‐value	AOR (95% CI)	*p*‐value
Food security								
Overall	0.70 (0.52–0.93)	0.015	0.19 (0.12–0.31)	<0.001	0.76 (0.56–1.04)	0.084	0.71 (0.53–0.94)	0.016
Among non‐mothers	0.45 (0.26–0.78)	0.073	0.32 (0.13–0.82)	0.23	1.60 (0.74–3.50)	0.037	0.63 (0.39–1.03)	0.597
Among adolescent mothers	0.81 (0.58–1.14)		0.17 (0.10–0.28)		0.66 (0.47–0.92)		0.74 (0.53–1.03)	
Among HIV‐uninfected non‐mothers	0.77 (0.25–2.38)	0.079	0.18 (0.03–1.11)	0.68	3.64 (0.82–16.05)	0.258	0.84 (0.36–1.97)	0.403
Among non‐mothers living with HIV	0.36 (0.19–0.68)		0.40 (0.13–1.20)		1.08 (0.43–2.71)		0.54 (0.30–0.99)	
Among HIV‐uninfected adolescent mothers	0.67 (0.46–1.00)		0.11 (0.06–0.21)		0.70 (0.46–1.06)		0.72 (0.48–1.07)	
Among adolescent mothers living with HIV	1.21 (0.65–2.27)		0.42 (0.17–1.09)		0.61 (0.35–1.05)		0.79 (0.44–1.43)	

	Sex on substances		Alcohol use		Not in education/employment			
	AOR (95% CI)	*p*‐value	AOR (95% CI)	*p*‐value	AOR (95% CI)	*p*‐value		
Food security								
Overall	0.57 (0.37–0.87)	0.010	0.65 (0.42–1.00)	0.048	0.53 (0.41–0.71)	<0.001		
Among non‐mothers	0.82 (0.32–2.08)	0.375	1.05 (0.54–2.07)	0.058	0.48 (0.29–0.77)	0.585		
Among adolescent mothers	0.51 (0.32–0.82)		0.45 (0.25–0.79)		0.56 (0.40–0.78)			
Among HIV‐uninfected non‐mothers	0.98 (0.20–4.86)	0.773	2.32 (0.55–9.70)	0.419	0.35 (0.15–0.83)	0.983		
Among non‐mothers living with HIV	0.73 (0.22–2.41)		0.75 (0.35–1.61)		0.55 (0.30–1.00)			
Among HIV‐uninfected adolescent mothers	0.51 (0.28–0.91)		0.51 (0.24–1.07)		0.51 (0.35–0.73)			
Among adolescent mothers living with HIV	0.53 (0.24–1.18)		0.37 (0.15–0.93)		0.78 (0.40–1.51)			

Note: *n* = 1638. For sub‐group analyses, we report Wald test *p*‐values for the interaction term. Multivariable models adjust for participant socio‐demographic characteristics: HIV status; age; relationship status; parental monitoring; rural/urban household location; informal housing type; number of people living in household; maternal orphanhood; and paternal orphanhood. Clustering of multiple outcomes within individuals is accounted for using the GEE method.

Abbreviations: AOR, adjusted odds ratio; CI, confidence interval; HIV, human immunodeficiency virus; OR, odds ratio.

### 3.4 Adjusted probabilities of HIV risk behaviours conditional on food security

**Figure 2:**
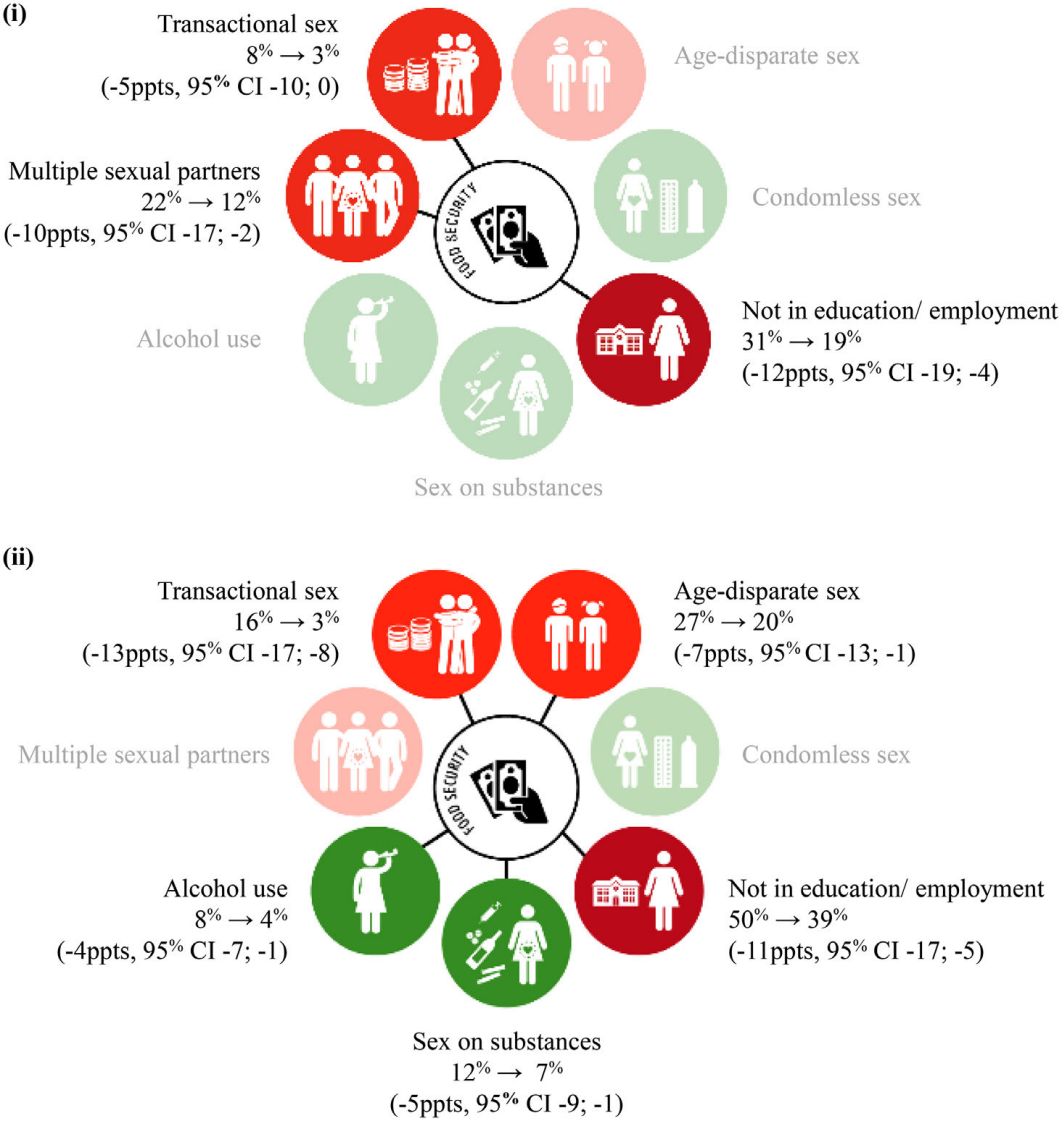
Adjusted probabilities and risk differences for HIV risk behaviours among (i) non‐mothers and (ii) adolescent mothers. Note: Percentages joined by an arrow are adjusted probabilities for the scenarios, (i) experiencing no food security and (ii) experiencing food security. Data in the brackets are adjusted probability differences and 95% confidence intervals. Green circles correspond to outcomes related to SDG 3: Good Health and Wellbeing. Red circles correspond to outcomes related to 4: Quality Education. Orange circles correspond to outcomes related to SDG 5: Gender Equality. Circles are faded when there was no evidence of an association between food security and the specific HIV risk behaviour. Adjusted probabilities were estimated with all covariates at observed values. The values used to build Figure 2 are provided in Table S5. Abbreviations: CI, confidence interval; HIV, human immunodeficiency virus; ppts, percentage points.

Probability differences contrasting adjusted probabilities of engaging in HIV risk behaviours for the scenarios of (1) not experiencing food security and (2) experiencing food security are shown in Figure 2 and Table S5. For *non‐mothers*, food security was associated with lower probability of multiple sexual partners (−10 percentage point [ppts], 95% CI = −17 ppts; −2 ppts); transactional sex (−5 ppts, 95% CI = −10 ppts; −0 ppts); and not in education/employment (−12 ppts, 95% CI = −19 ppts; −4 ppts). For *adolescent mothers*, food security was associated with lower probability of transactional sex (−13 ppts, 95% CI = −17 ppts; −8 ppts); age‐disparate sex (−7 ppts, 95% CI = −13 ppts; −1 ppts); sex on substances (−5 ppts, 95% CI = −9 ppts; −1 ppts); alcohol use (−4 ppts, 95% CI = −7 ppts; −1 ppts); and not in education/employment (−11 ppts, 95% CI = −17 ppts; −5 ppts).

### 3.5 Sensitivity analysis

Dropping non‐mothers without a first sexual experience, since all adolescent mothers would have had a sexual experience, results were the same except that there was no evidence of an association between adolescent motherhood and multiple sexual partners, or sex on substances, and that in adolescent mothers, there was evidence that food security may additionally be associated with lower odds of condomless sex (Tables S6 and S7).

## 4 DISCUSSION

Findings from this study provide new knowledge around the drivers of HIV infection and onwards transmission (both vertical and horizontal) among AGYW in Southern Africa experiencing high rates of adversity, motherhood and HIV. Adolescent motherhood was associated with lower odds of alcohol use, but higher odds of multiple sexual partners, age‐disparate sex, condomless sex, sex on substances and not being in education/employment. We also observed that the combination of adolescent motherhood and living with HIV was associated with significantly higher odds of age disparate sex and not being in education/employment as compared to the combination of adolescent motherhood and not living with HIV. Focusing on ways to reduce HIV risk, we established food security to be associated with lower odds of three HIV risk behaviours in non‐mothers and lower odds of five HIV risk behaviours in adolescent mothers. We observed no differences in the associations between food security and HIV risk behaviours by HIV status either among adolescent mothers or among non‐mothers.

Our findings align with new evidence that adolescent mothers are poorer and at higher risk of HIV infection across 10 high‐HIV burden countries in Eastern and Southern Africa [16], and separately, that food security and adolescent motherhood are strongly linked in the same region [17, 45, 46]. Economic constraints are a key driver of HIV risk behaviours, including multiple sexual partners, age‐disparate sex and school dropout among adolescent girls [47, 48]. In this study, associations between adolescent motherhood and these behaviours may be linked to educational delay and childcare responsibilities that limit opportunities for education and employment [49, 50]. There is also evidence that familial rejection due to stigma exacerbates adolescent mothers’ dependence on male partners [17]. Our observation that food security is likely to be associated with lower probability of HIV risk behaviours in both non‐mothers and adolescent mothers is consistent with previously observed links between hunger, risky sex and HIV infection [51], and quantitative evaluations of economic‐strengthening interventions [52].

There is an urgent need to accelerate HIV prevention among AGYW, especially following increased rates of food insecurity and adolescent pregnancy linked to the Covid‐19 pandemic [22, 23, 53]. Evidence from our study highlights the need for, and potential of, scalable social protection initiatives to improve food security in the African region. These include cash transfers, in‐kind transfers, such as school meals and school bursaries, and income‐generating programmes [54]. Where initiatives already exist, referral systems can be used to link frequently used services (e.g. schools and sexual and reproductive health clinics) with social protection. Services can also be expanded, for example, free school meals that are mostly offered in primary school could be expanded to secondary schools. Importantly, with rates of school enrolment so low among adolescent mothers, efforts will be needed to facilitate this groups’ access to school‐based programmes [55]. Additionally, the lack of evidence in our study for a stronger relationship between food security and HIV risk among adolescent mothers, particularly those living with HIV, also highlights a need for additional forms of support beyond social protection if we are to address the disproportionately high rates of most HIV risk behaviours in this population. For example, affordable childcare may be essential for supporting adolescent mothers to attend education and/or income‐generating programs [56].

The study has strengths and limitations. To our knowledge, it is the first known survey to evaluate the relationship between food security and HIV risk behaviours among adolescent mothers and non‐mothers in sub‐Saharan Africa. The study's exhaustive sampling strategy aimed to ensure that even the most vulnerable adolescent mothers were recruited. Inclusion of participants uninfected and living with HIV enabled us to consider how the relationship between adolescent motherhood and risk behaviours was moderated by this third variable. For limitations, the study's cross‐sectional design precludes causal claim for observed associations. Self‐report items may also be subject to social desirability response bias. Although we aimed to minimize this by conducting interviews in a location of the adolescent's choice and by using audio mobile‐assisted self‐interviewing for sensitive topics. Although rates of risk behaviours reported in the study were similar to those found in South African population‐based studies [57], because the MW and HEY BABY cohorts oversampled adolescents living with HIV, the prevalence of some risk behaviours and their associations with predictors should only be interpreted disaggregated by HIV status. Around 10% of mothers had conceived 12–13 months previously, and so there may be some crossover between self‐reported condomless sex and conception for this subgroup. Our measure of food security, which asked about having enough food at home, may have missed certain dimensions of this construct, including dietary diversity and nutritional absorption. Future research would valuably include nutrition assessments, such as BMI‐for‐age.

For the future, further longitudinal studies around adolescent motherhood, food security and HIV risk will be required to unpack the cross‐sectional relationships observed in this study. In particular, we need to understand further the relative risk of horizontal HIV infection for AGYW prior, during, and after pregnancy and the influence of socio‐economic and behavioural risk factors during each of these phases. Further research would also be valuable around how adolescent mothers’ economic dependence on male partners shapes their sexual relationships. There should also be more research examining the linkages of food security with pre‐exposure prophylaxis (PrEP) use for adolescent mothers not living with HIV, and ART‐adherence and viral load suppression among those living with HIV. Absence of evidence linking food security and condomless sex in either non‐mothers or adolescent mothers highlights the need for further research to identify interventions that act on this outcome in these high‐risk groups [58]. Finally, further research should also examine food security and HIV risk in contexts of high child marriage, as in line with national statistics, fewer than 1% of AGYW in this study were married [59, 60, 61].

## 5 CONCLUSIONS

Within the high‐risk group of AGYW in sub‐Saharan Africa, adolescent mothers are a priority group experiencing multiple vulnerabilities to HIV infection and transmission. Promoting access to sufficient food is likely to mitigate HIV risk pathways among AGYW, with greater impacts for adolescent mothers.

## COMPETING INTERESTS

Nothing to declare.

## AUTHORS’ CONTRIBUTIONS

LC: Funding acquisition, conceptualization, project administration, supervision, writing—original draft, review and editing; WER: Conceptualization, data curation, formal analysis, methodology, project administration, writing—original draft, review and editing; ET: Funding acquisition, conceptualization, supervision, writing—review and editing; MO: Conceptualization, writing—review and editing; MI: Conceptualization, writing—review and editing; NL: Conceptualization, writing—review and editing; CK: Conceptualization, writing—review and editing; RY: Conceptualization, writing—review and editing; NX, KR, MS, SB, GG and MM: Conceptualization, review and editing. LS: Conceptualization, writing—review and editing.

## FUNDING

This study was supported by the Fogarty International Center, National Institute on Mental Health, National Institutes of Health (K43TW011434); the Claude Leon Foundation F08 559/C; the UK Economic and Social Research Council (ES/J500112/1 and ES/R501037/1); the Regional Inter‐Agency Task Team for Children Affected by AIDS – Eastern and Southern Africa (RIATT‐ESA); the International AIDS Society through the CIPHER grant (155‐Hod; 2018 and 625‐TOS); Evidence for HIV Prevention in Southern Africa (EHPSA), a UK aid programme managed by Mott MacDonald; the European Research Council (ERC) under the European Union's Horizon 2020 research and innovation programme (grant agreement no. 771468); Research England (0005218); Janssen Pharmaceutica N.V., part of the Janssen Pharmaceutical Companies of Johnson & Johnson; the Medical Research Council (MRC) and the Department of Health Social Care (DHSC) through its National Institutes of Health Research (NIHR) (MR/R022372/1); the Nuffield Foundation, but the views expressed are those of the authors and not necessarily the Foundation; the John Fell Fund (161/033); the Philip Leverhulme Trust (PLP‐2014‐095); the University of Oxford's ESRC Impact Acceleration Account (1602‐KEA‐189 and K1311‐KEA‐004); UCL's HelpAge International; UNFPA South Africa; World Food Programme; UNICEF Eastern and Southern Africa Office (UNICEF‐ESARO); UKRI GCRF Accelerating Achievement for Africa's Adolescents (Accelerate) Hub (grant ref: ES/S008101/1); Wellspring Philanthropic Fund (grant no. 16204); Oak Foundation/GCRF “Accelerating Violence Prevention in Africa” (R46194/AA001 and OFIL‐20‐057).

## Supporting information


**Figure S1**. Flow chart of participants included in the study.

**Table S1**. STROBE checklist for cross‐sectional studies.

**Table S2**. Summary of missing values in study variables.

**Table S3**. Correlations across HIV risk behaviour outcomes by adolescent motherhood.

**Table S4**. Univariable associations between adolescent motherhood and HIV risk behaviours.

**Table S5**. Adjusted probabilities and probability differences for HIV risk behaviours amongst non‐mothers, and adolescent mothers. Adjusted probabilities were estimated for the scenarios, i) experiencing no food security and ii) experiencing food security. Adjusted probabilities were estimated with all covariates at observed values.

**Table S6**. Multivariate multivariable associations between adolescent motherhood and HIV risk behaviours, amongst non‐mothers and adolescent mothers with a first sexual experience accounting for correlation between outcomes using the GEE method.

**Table S7**. Multivariate multivariable associations between food security and HIV risk behaviours, amongst non‐mothers and adolescent mothers with a first sexual experience accounting for correlation between outcomes using the GEE method.

## Data Availability

The data that support the findings of this study are available from the corresponding author upon reasonable request.
