# Supplementary figures and images for: Food security reduces multiple HIV infection risks for high‐vulnerability adolescent mothers and non‐mothers in South Africa: a cross‐sectional study
Source: J Int AIDS Soc. 2022 Aug 25;25(8):e25928. doi: 10.1002/jia2.25928 (PMC9411725; doi:10.1002/jia2.25928)

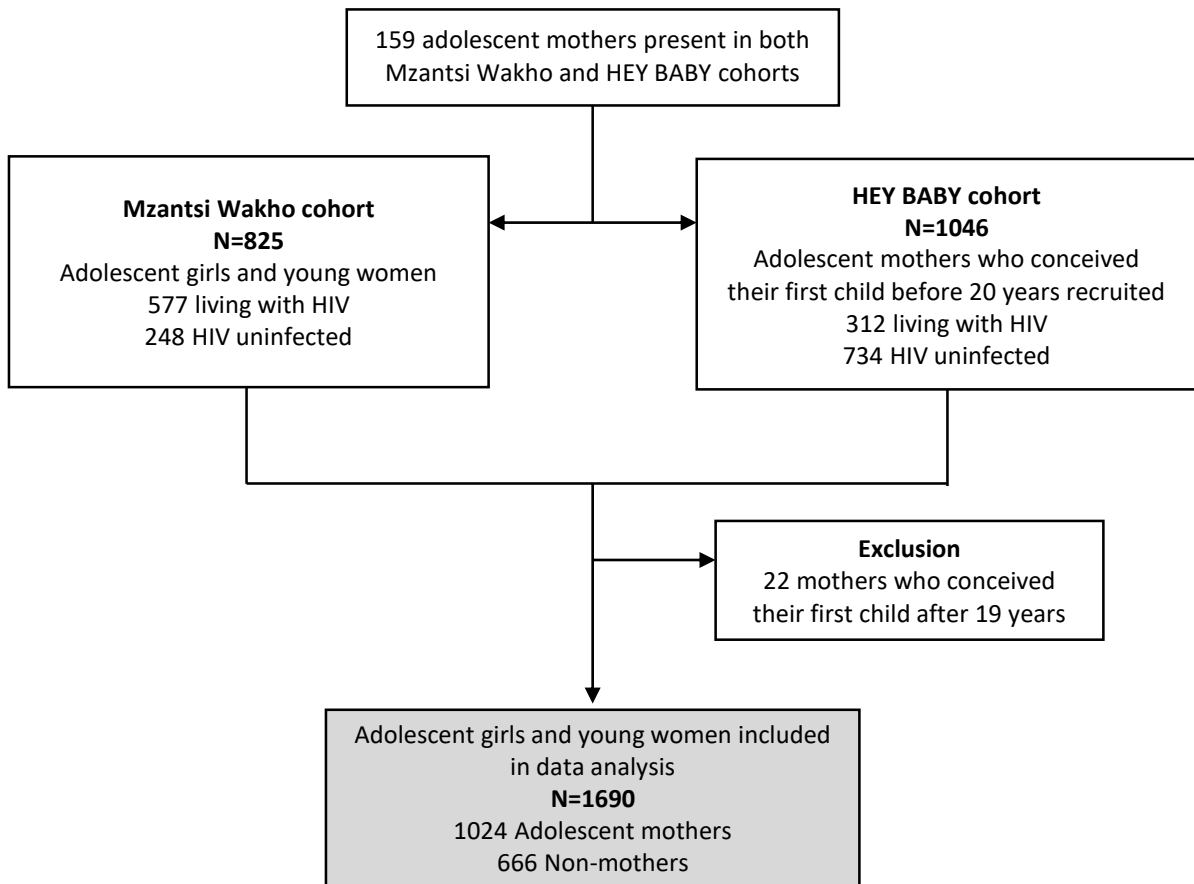

**S1 Figure. Flow chart of participants included in the study.**

Supplement: Supplementary file 1 — Figure S1. Flow chart of participants included in the study. [file JIA2-25-e25928-s004.pdf]
