# Supplementary material for: Food security reduces multiple HIV infection risks for high‐vulnerability adolescent mothers and non‐mothers in South Africa: a cross‐sectional study
Source: J Int AIDS Soc. 2022 Aug 25;25(8):e25928. doi: 10.1002/jia2.25928 (PMC9411725; doi:10.1002/jia2.25928)
Supplement: Supplementary file 3 — Table S2. Summary of missing values in study variables. [file JIA2-25-e25928-s002.docx]

**S2 Table. Summary of missing values in study variables.**

|  | **Missing values N=1690** |
| --- | --- |
| Age | 3 (0) |
| HIV status | 0 (0) |
| Relationship status | 0 (0) |
| Experience of parental monitoring | 0 (0) |
| Rural | 2 (0) |
| Informal house | 24 (1) |
| Household size | 0 (0) |
| Maternal orphan | 0 (0) |
| Paternal orphan | 0 (0) |
| Food security | 0 (0) |
| Multiple sexual partners | 0 (0) |
| Transactional sex | 0 (0) |
| Age-disparate sex | 24 (1) |
| Condomless sex | 0 (0) |
| Sex on substances | 0 (0) |
| Alcohol use | 0 (0) |
| Not in education/ employment | 0 (0) |

Abbreviations: HIV, human immunodeficiency virus.
