# Supplementary material for: Food security reduces multiple HIV infection risks for high‐vulnerability adolescent mothers and non‐mothers in South Africa: a cross‐sectional study
Source: J Int AIDS Soc. 2022 Aug 25;25(8):e25928. doi: 10.1002/jia2.25928 (PMC9411725; doi:10.1002/jia2.25928)
Supplement: Supplementary file 4 — Table S3. Correlations across HIV risk behaviour outcomes by adolescent motherhood. [file JIA2-25-e25928-s003.docx]

**S3 Table. Correlations across HIV risk behaviour outcomes by adolescent motherhood.**

|  | **n (%)** | **Correlations** | | | | | | |
| --- | --- | --- | --- | --- | --- | --- | --- | --- |
|  |  | **1** | **2** | **3** | **4** | **5** | **6** | **7** |
| **Non-mothers (n=666)** |  |  |  |  |  |  |  |  |
| 1. Multiple sexual partners | 80 (12) | 1.00 |  |  |  |  |  |  |
| 2. Transactional sex | 21 (3) | 0.30 | 1 |  |  |  |  |  |
| 3. Age-disparate sex† | 47 (7) | 0.30 | 0.05 | 1.00 |  |  |  |  |
| 4. Condomless sex | 90 (14) | 0.37 | 0.10 | 0.26 | 1.00 |  |  |  |
| 5. Sex on substances | 26 (4) | 0.28 | 0.05 | 0.10 | 0.35 | 1.00 |  |  |
| 6. Alcohol | 55 (8) | 0.19 | -0.02 | 0.18 | 0.25 | 0.16 | 1.00 |  |
| 7. Not in education/ employment | 110 (17) | 0.20 | 0.13 | 0.23 | 0.31 | 0.12 | 0.15 | 1.00 |
| **Adolescent mothers (N=1024)** |  |  |  |  |  |  |  |  |
| 1. Multiple sexual partners | 270 (26) | 1.00 |  |  |  |  |  |  |
| 2. Transactional sex | 79 (8) | 0.04 | 1.00 |  |  |  |  |  |
| 3. Age-disparate sex‡ | 236 (23) | 0.12 | 0.13 | 1.00 |  |  |  |  |
| 4. Condomless sex | 671 (66) | 0.03 | -0.01 | 0.08 | 1.00 |  |  |  |
| 5. Sex on substances | 101 (10) | 0.21 | 0.04 | 0.05 | 0.05 | 1.00 |  |  |
| 6. Alcohol | 62 (6) | 0.05 | -0.04 | 0.03 | 0.01 | 0.25 | 1.00 |  |
| 7. Not in education/ employment | 462 (45) | 0.12 | -0.03 | 0.11 | 0.03 | 0.05 | 0.06 | 1.00 |

Coefficients are spearman correlations.

†5 non-mothers are missing information on age-disparate sex.

‡19 adolescent mothers are missing information on age-disparate sex.
