# Supplementary material for: Food security reduces multiple HIV infection risks for high‐vulnerability adolescent mothers and non‐mothers in South Africa: a cross‐sectional study
Source: J Int AIDS Soc. 2022 Aug 25;25(8):e25928. doi: 10.1002/jia2.25928 (PMC9411725; doi:10.1002/jia2.25928)
Supplement: Supplementary file 5 — Table S4. Univariable associations between adolescent motherhood and HIV risk behaviours. [file JIA2-25-e25928-s008.docx]

**S4 Table. Univariable associations between adolescent motherhood and HIV risk behaviours.**

|  | **Multiple sexual partners** | | **Transactional sex** | | **Age-disparate sex†** | | **Condomless sex** | | |
| --- | --- | --- | --- | --- | --- | --- | --- | --- | --- |
|  | OR (95% CI) | p-value | OR (95% CI) | p-value | OR (95% CI) | p-value | OR (95% CI) | p-value |  |
| Adolescent motherhood | 2.62 (2.00-3.44) | <0.001 | 2.57 (1.57-4.20) | <0.001 | 4.01 (2.88-5.58) | <0.001 | 12.17 (9.41-15.73) | <0.001 |  |
|  | **Sex on substances** | | **Alcohol** | | **Not in education/ employment** | |  |  |  |
|  | OR (95% CI) | p-value | OR (95% CI) | p-value | OR (95% CI) | p-value |  |  |  |
| Adolescent motherhood | 2.69 (1.73-4.19) | <0.001 | 0.72 (0.49-1.04) | 0.08 | 4.16 (3.27-5.28) | <0.001 |  |  |  |

N=1690.

†24 participants are missing information on age-disparate sex, including five non-mothers, and 19 adolescent mothers.

Abbreviations: OR, odds ratio; CI, confidence interval.
