# Supplementary material for: Food security reduces multiple HIV infection risks for high‐vulnerability adolescent mothers and non‐mothers in South Africa: a cross‐sectional study
Source: J Int AIDS Soc. 2022 Aug 25;25(8):e25928. doi: 10.1002/jia2.25928 (PMC9411725; doi:10.1002/jia2.25928)
Supplement: Supplementary file 6 — Table S5. Adjusted probabilities and probability differences for HIV risk behaviours amongst non‐mothers, and adolescent mothers. Adjusted probabilities were estimated for the scenarios, i) experiencing no food security and ii) experiencing food security. Adjusted probabilities were estimated with all covariates at observed values. [file JIA2-25-e25928-s005.docx]

**S5 Table. Adjusted probabilities and probability differences for HIV risk behaviours amongst non-mothers, and adolescent mothers.** Adjusted probabilities were estimated for the scenarios, i) experiencing no food security and ii) experiencing food security. Adjusted probabilities were estimated with all covariates at observed values.

|  | **Multiple sexual partners** | | **Transactional sex** | | **Age-disparate sex** | | **Condomless sex** | |
| --- | --- | --- | --- | --- | --- | --- | --- | --- |
|  | **Adjusted probability  (95% CI)** | **Probability difference (95% CI)** | **Adjusted probability  (95% CI)** | **Probability difference (95% CI)** | **Adjusted probability  (95% CI)** | **Probability difference (95% CI)** | **Adjusted probability  (95% CI)** | **Probability difference (95% CI)** |
| **Non-mothers** |  |  |  |  |  |  |  |  |
| Food security |  |  |  |  |  |  |  |  |
| No | 21.54 (14.73; 28.34) | - | 7.57 (0.03; 12.31) | - | 7.28 (2.78; 11.78) | - | 22.92 (15.93; 29.91) | - |
| Yes | 11.88 (8.54; 15.23) | -9.65 (-16.94; -2.37) | 2.63 (0.91; 4.35) | -4.94 (-9.85; -0.03) | 11.05 (7.30; 14.79) | 3.77 (-1.98; 9.51) | 16.19 (12.32; 20.07) | -6.73 (-14.33; 0.88) |
| **Adolescent mothers** |  |  |  |  |  |  |  |  |
| Food security |  |  |  |  |  |  |  |  |
| No | 25.24 (20.53; 29.94) | - | 15.97 (11.48; 20.47) | - | 27.76 (22.24; 33.27) | - | 64.43 (58.22; 70.65) | - |
| Yes | 21.98 (19.10; 24.86) | -3.26 (-8.64; 2.13) | 3.30 (2.04; 4.57) | -12.67 (-17.25; -8.08) | 20.58 (17.44; 23.71) | -7.18 (-13.16; -1.20) | 57.95 (54.12; 61.78) | -6.48 (-13.51; 0.54) |
|  | **Sex on substances** | | **Alcohol use** | | **Not in education/ employment** | |  |  |
|  | **Adjusted probability  (95% CI)** | **Probability difference (95% CI)** | **Adjusted probability  (95% CI)** | **Probability difference (95% CI)** | **Adjusted probability  (95% CI)** | **Probability difference (95% CI)** |  |  |
| **Non-mothers** |  |  |  |  |  |  |  |  |
| Food security |  |  |  |  |  |  |  |  |
| No | 5.51 (1.73; 9.28) | - | 9.83 (5.34; 14.31) | - | 30.78 (23.54; 38.03) | - |  |  |
| Yes | 4.61 (2.37; 6.85) | -0.90 (-5.23; 3.43) | 10.25 (7.00; 13.51) | 0.42 (-4.99; 5.84) | 19.24 (15.00; 23.48) | -11.54 (-19.41; -3.67) |  |  |
| **Adolescent mothers** |  |  |  |  |  |  |  |  |
| Food security |  |  |  |  |  |  |  |  |
| No | 11.92 (8.36; 15.48) | - | 8.33 (5.30; 11.35) | - | 49.54 (43.86; 55.22) | - |  |  |
| Yes | 6.86 (5.16; 8.56) | -5.07 (-8.97; -1.16) | 4.15 (2.76; 5.54) | -4.17 (-7.46; -0.89) | 38.65 (35.29; 42.00) | -10.89 (-17.09; -4.69) |  |  |

Abbreviations: CI, confidence interval.
