# Supplementary material for: Food security reduces multiple HIV infection risks for high‐vulnerability adolescent mothers and non‐mothers in South Africa: a cross‐sectional study
Source: J Int AIDS Soc. 2022 Aug 25;25(8):e25928. doi: 10.1002/jia2.25928 (PMC9411725; doi:10.1002/jia2.25928)
Supplement: Supplementary file 7 — Table S6. Multivariate multivariable associations between adolescent motherhood and HIV risk behaviours, amongst non‐mothers and adolescent mothers with a first sexual experience accounting for correlation between outcomes using the GEE method. [file JIA2-25-e25928-s007.docx]

**S6 Table. Multivariate multivariable associations between adolescent motherhood and HIV risk behaviours, amongst non-mothers and adolescent mothers with a first sexual experience accounting for correlation between outcomes using the GEE method.**

|  | **Multiple sexual partners** | | **Transactional sex** | | **Age-disparate sex** | | **Condomless sex** | |
| --- | --- | --- | --- | --- | --- | --- | --- | --- |
|  | AOR (95% CI) | p-value | AOR (95% CI) | p-value | AOR (95% CI) | p-value | AOR (95% CI) | p-value |
| **Adolescent motherhood** |  |  |  |  |  |  |  |  |
| Overall | 1.06 (0.74-1.5) | 0.756 | 1.07 (0.60-1.93) | 0.816 | 1.67 (1.12-2.49) | 0.013 | 3.39 (2.46-4.67) | <0.001 |
| Among HIV uninfected AGYW | 1.22 (0.71-2.12) | 0.480 | 1.39 (0.55-3.53) | 0.485 | 0.91 (0.53-1.59) | 0.008 | 3.90 (2.45-6.21) | 0.409 |
| Among AGYW living with HIV | 0.95 (0.60-1.50) |  | 0.91 (0.41-1.99) |  | 2.52 (1.52-4.19) |  | 2.99 (1.94-4.62) |  |
|  | **Sex on substances** | | **Alcohol use** | | **Not in education/ employment** | |  |  |
|  | AOR (95% CI) | p-value | AOR (95% CI) | p-value | AOR (95% CI) | p-value |  |  |
| **Adolescent motherhood** |  |  |  |  |  |  |  |  |
| Overall | 1.26 (0.75-2.13) | 0.377 | 0.31 (0.20-0.50) | <0.001 | 2.70 (1.88-3.87) | <0.001 |  |  |
| Among HIV uninfected AGYW | 1.11 (0.53-2.31) | 0.632 | 0.25 (0.13-0.50) | 0.367 | 1.43 (0.88-2.32) | 0.001 |  |  |
| Among AGYW living with HIV | 1.41 (0.7-2.84) |  | 0.38 (0.21-0.69) |  | 4.22 (2.65-6.73) |  |  |  |

N=1242.

For sub-group analyses, we report Wald test p-values for the interaction term.

Multivariable models adjust for participant characteristics: age, HIV status, relationship status, parental monitoring, rural/urban household location, informal housing type, number of people living in household, maternal orphanhood, paternal orphanhood, and food security.

Abbreviations: AOR, adjusted odds ratio; CI, confidence interval; HIV, human immunodeficiency virus; AGYW, adolescent girls and young women.
