# Supplementary material for: Food security reduces multiple HIV infection risks for high‐vulnerability adolescent mothers and non‐mothers in South Africa: a cross‐sectional study
Source: J Int AIDS Soc. 2022 Aug 25;25(8):e25928. doi: 10.1002/jia2.25928 (PMC9411725; doi:10.1002/jia2.25928)
Supplement: Supplementary file 8 — Table S7. Multivariate multivariable associations between food security and HIV risk behaviours, amongst non‐mothers and adolescent mothers with a first sexual experience accounting for correlation between outcomes using the GEE method. [file JIA2-25-e25928-s001.docx]

**S7 Table. Multivariate multivariable associations between food security and HIV risk behaviours, amongst non-mothers and adolescent mothers with a first sexual experience accounting for correlation between outcomes using the GEE method.**

|  | **Multiple sexual partners** | | **Transactional sex** | | **Age-disparate sex** | | **Condomless sex** | | |
| --- | --- | --- | --- | --- | --- | --- | --- | --- | --- |
|  | AOR (95% CI) | p-value | AOR (95% CI) | p-value | AOR (95% CI) | p-value | AOR (95% CI) | p-value |  |
| **Food security** |  |  |  |  |  |  |  |  |  |
| Overall | 0.69 (0.51-0.92) | 0.012 | 0.19 (0.12-0.30) | <0.001 | 0.75 (0.55-1.01) | 0.062 | 0.67 (0.50-0.89) | 0.007 |  |
| Among non-mothers | 0.42 (0.22-0.80) | 0.084 | 0.34 (0.13-0.91) | 0.18 | 1.71 (0.77-3.82) | 0.025 | 0.58 (0.32-1.06) | 0.587 |  |
| Among adolescent mothers | 0.79 (0.57-1.10) |  | 0.16 (0.10-0.28) |  | 0.64 (0.46-0.89) |  | 0.70 (0.51-0.96) |  |  |
| Among HIV uninfected non-mothers | 0.56 (0.16-1.99) | 0.26 | 0.14 (0.02-0.91) | 1.00 | 2.94 (0.63-13.66) | 0.537 | 0.51 (0.18-1.43) | 0.811 |  |
| Among non-mothers living with HIV | 0.37 (0.17-0.81) |  | 0.50 (0.16-1.56) |  | 1.36 (0.52-3.56) |  | 0.64 (0.31-1.35) |  |  |
| Among HIV uninfected adolescent-mothers | 0.67 (0.45-0.98) |  | 0.11 (0.06-0.20) |  | 0.68 (0.45-1.03) |  | 0.69 (0.47-1.01) |  |  |
| Among adolescent-mothers living with HIV | 1.14 (0.63-2.08) |  | 0.40 (0.16-1.03) |  | 0.58 (0.34-1.00) |  | 0.73 (0.42-1.28) |  |  |
|  | **Sex on substances** | | **Alcohol use** | | **Not in education/ employment** | |  |  |  |
|  | AOR (95% CI) | p-value | AOR (95% CI) | p-value | AOR (95% CI) | p-value |  |  |  |
| **Food security** |  |  |  |  |  |  |  |  |  |
| Overall | 0.57 (0.37-0.87) | 0.010 | 0.62 (0.40-0.96) | 0.033 | 0.51 (0.39-0.68) | <0.001 |  |  |  |
| Among non-mothers | 0.85 (0.34-2.11) | 0.327 | 1.01 (0.50-2.04) | 0.078 | 0.37 (0.20-0.68) | 0.238 |  |  |  |
| Among adolescent mother | 0.51 (0.31-0.81) |  | 0.45 (0.26-0.78) |  | 0.56 (0.41-0.77) |  |  |  |  |
| Among HIV uninfected non-mothers | 0.82 (0.16-4.09) | 0.990 | 1.94 (0.45-8.35) | 0.543 | 0.22 (0.07-0.66) | 0.647 |  |  |  |
| Among non-mothers living with HIV | 0.85 (0.27-2.68) |  | 0.73 (0.32-1.68) |  | 0.47 (0.22-1.00) |  |  |  |  |
| Among HIV uninfected adolescent-mothers | 0.51 (0.28-0.90) |  | 0.51 (0.25-1.07) |  | 0.51 (0.36-0.73) |  |  |  |  |
| Among adolescent-mothers living with HIV | 0.52 (0.23-1.15) |  | 0.37 (0.15-0.89) |  | 0.77 (0.41-1.45) |  |  |  |  |

N=1242.

For sub-group analyses, we report Wald test p-values for the interaction term.

Multivariable models adjust for participant characteristics: age, HIV status, relationship status, parental monitoring, rural/urban household location, informal housing type, number of people living in household, maternal orphanhood, paternal orphanhood, and food security.

Abbreviations: AOR, adjusted odds ratio; CI, confidence interval; HIV, human immunodeficiency virus.
